# Correlations between muscle-tendon parameters and acceleration ability in 20 m sprints

**DOI:** 10.1371/journal.pone.0213347

**Published:** 2019-03-08

**Authors:** Andrea Monte, Paola Zamparo

**Affiliations:** Department of Neuroscience, Biomedicine and Movement Sciences, University of Verona, Verona, Italy; Universidad Autonoma de Madrid, SPAIN

## Abstract

In this study we investigated the relationships between muscle-tendon parameters and average/peak values of velocity, force and power in sprint running focusing on the acceleration phase. Eighteen male sprinters (100 m PB: 10.66±0.51 s) participated to the study. Instantaneous values of horizontal velocity (v) were recorded by means of a radar and instantaneous values of force (F) and power (P) were calculated based on these data. Muscle thickness, fascicle length and pennation angle of knee extensors and plantar flexors, as well as Achilles tendon length and CSA, were measured by means of ultrasonography. In the first 20 m of the sprint average and peak speed were 6.31±0.59 and 8.88±0.98 m·s^-1^, respectively; force was highest at the start of the sprint (F_peak_ = 10.02±1.43 N·kg^-1^) and power peaked about 1 s after the start (26.64±5.99 W·kg^-1^). Muscle-tendon parameters showed stronger correlations with peak values of power (R range: 0.81–0.92), force (R range: 0.56–0.84) and speed (R range: 0.53–0.85) than with average values of velocity over the 20 m distance (R range: 0.41–0.61) (*R* <0.47 = NS; *R* >0.71 = *P* < .001). These data underline that the influence of muscle tendon parameters on sprint performance could be better appreciated when peak values of power can be calculated rather than by considering the simple measure of average velocity (e.g. distance/time).

## Introduction

Sprint performance (e.g. in a 100 m race) is determined by the ability to accelerate rapidly, by the magnitude of maximal velocity and by the ability to maintain this velocity up to the end of the race [1]; the ability to accelerate rapidly in the first steps of a sprint is what separates an elite sprinter from a merely good one [2]; indeed, best sprinters exert larger propulsive forces (relative horizontal impulses) in the sprint running acceleration phase [3,4,5]. The ability to accelerate is a key parameter also in many team sports and the goal of many training programs.

The ability to generate large forward accelerations is thus related to the capability to produce high amounts of horizontal external force over the entire acceleration phase: the changes in velocity during a sprint running acceleration phase are indeed accompanied by changes in force production [4–5]. As an example Rabita et al. [6] have shown that, in the first 30 m of a 100 m race the increase in running velocity is mirrored by a decrease in horizontal force; as a result, power output, which is recognized to be the major determinant of sprint performance and acceleration ability [5,6,7], is maximal in the first steps of a sprint.

At the muscle level, force, velocity and power are influenced by fibre type distribution and architecture: i) fast contracting fibres can shorten up to 2–3 times faster than slow contracting ones; ii) muscles with larger cross sectional area (CSA) generate larger tensions and peak isometric forces (this is, as an example, the case of pennate muscles); iii) muscles with (relatively) longer fibres (e.g. fusiform, non-pennate, muscles) can contract more rapidly and generate peak power at a higher velocity [8,9,10,11,12,13].

For these reasons the relationship between sprint performance and muscle architecture was investigated in the literature. However, this was done in just few studies (to our knowledge) that related top running velocity, or personal best time in a 100 m event, with muscle-tendon characteristics. Kumagai et al. [14], reported a significant negative relationship between 100 m best time and fascicle length of vastus lateralis (VL, *R* = -0.43, *P* < .01), gastrocnemius medialis (GM, *R* = -0.44, *P* < .01) and lateralis (GL, *R* = -0.57, p < 0.01) whereas Abe et al. [15] reported a significant negative relationship between 100 m best time and fascicle length of VL (*R* = -0.51, *P* < .01) and GL (*R* = -0.44, *P* < .05) but not with GM (*R* = -0.22, NS). At the same time, Kumagai et al. [14] demonstrated significant positive relationships between 100 m sprint time and pennation angle of VL (R = 0.34, P < .05), GL (R = 0.46, P < .01) and GM (R = 0.37, P < .05). More recently, Kubo et al. [16], reported significant positive relationships between muscle thickness of knee extensors (*R* = 0.616, *P* < .05) and 100 m race time while they found no relationship with tendon stiffness, and elongation, of the knee extensor muscles (*R* = 0.194 and *R* = 0.249, NS, respectively). Stafilidis & Arampatzis [17] reported a negative relationship between maximal elongation of VL tendon and aponeurosis with 100 m sprint times (*R* = -0.57, *P* <0.01). Finally, Miyake et al. [18] reported no relationship between 100 m best time and quadriceps CSA (*R* = 0.265, NS) while they found a significant positive relationship between 100 m best time and knee extensors moment arm (*R* = 0.614, *P* < .001); interestingly, stronger relationships were found, in Miyake’s study [18], when correlating knee extensors moment arm with sprinting velocity (in the acceleration phase: *R* = 0.72) or maximal velocity (*R* = 0.62).

In conclusion, a strong correlation should be expected between muscle-tendon parameters and sprint performance (as was indeed the case in many of the cited studies). However, as indicated above, the capacity to exert force (and power) is an important determinant of performance in the first 20–30 m of a sprint; it is thus likely that muscle-tendon parameters will correlate better with measures of force (and power) in this phase than with measures of average speed (e.g. 100 m best time).

We thus decided to focus our attention on the acceleration phase (the phase of peak force and peak power production) and to investigate the correlations between muscle-tendon parameters (knee extensors, plantar flexors and Achilles tendon) and some of the parameters which determine sprint performance in this phase (power, force and velocity as well as the degree of curvature of the v vs. t relationship).

Our hypothesis was, therefore, that muscle-tendon characteristics would be more strongly related to peak rather than mean values of speed, force and power in the first 20 m of a sprint (as well as with the sprinter’s 100 m PB) since it is in the acceleration phase that the sprinters have to provide the largest values of force (and power).

## Materials and methods

### Subjects

Eighteen male sprinters participated in this study (age: 24.36±5.0 years; body mass: 74.5±5.92 kg; stature: 1.78±0.06 m); they were free from any type of injury (this was verified by means of an interview) and were asked to abstain from training in the 24 hours before the testing session. The current personal best times over the 100 m distance (100 m PB) in these sprinters (10.66±0.51 s; range 10.02–11.44 s) were attained in the competitive season preceding the experiments. All participants received written and oral instructions before the study and gave their written informed consent to the experimental procedure. The experimental protocol was approved by the Ethical Committee of Verona University and performed in accordance with the Helsinki Declaration.

### Experimental procedure

#### Muscle-tendon characteristics

Muscle-tendon parameters were measured in vivo using B-Mode ultrasound apparatus after 10 min of resting. The athletes remained in a supine position for the measurement of quadriceps muscles and in a prone position for the measurements of plantar-flexors muscle and Achilles tendon, with the muscles relaxed, the leg straight, (i.e. hip and knee extended) and the ankle flexed at 90°. A water-soluble gel was applied to the transducer to aid acoustic coupling and to reduce the deformations of the muscle and tendon. For each muscle, two images were captured and subsequently analysed with ImageJ software and Photoshop CS5 by the same operator, their average value was utilized in further analysis.

**Fig 1:**
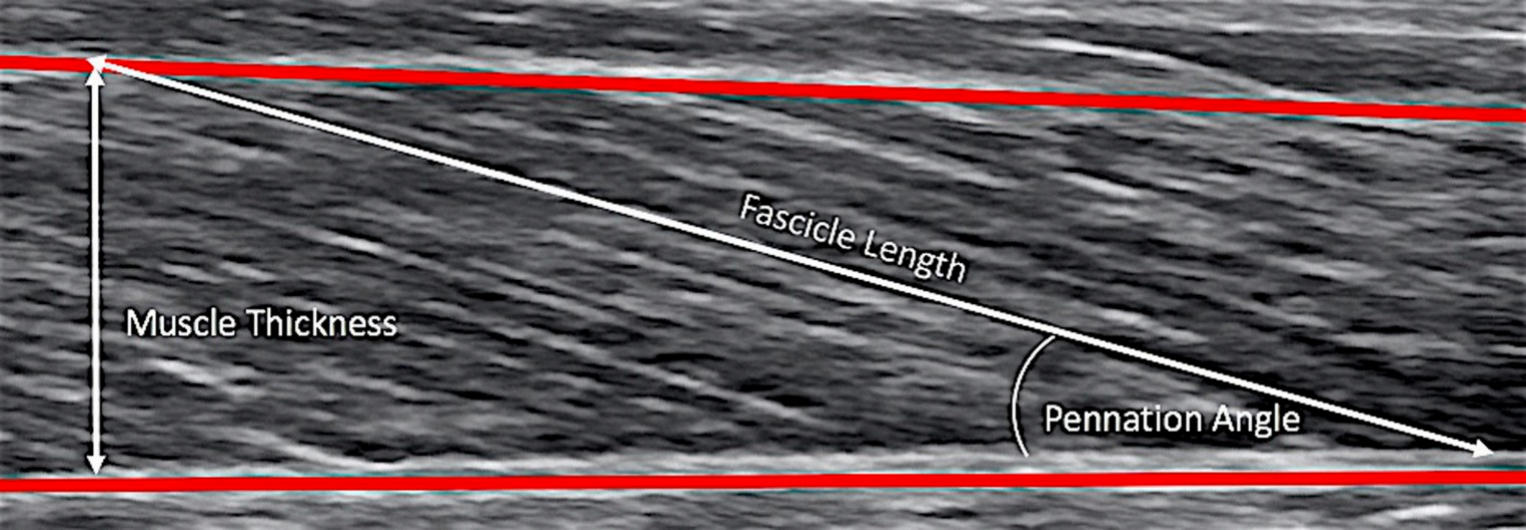
Ultrasonographic (longitudinal) image for measuring muscle thickness, pennation angle and fascicle length in vastus lateralis.

Muscle thickness of the knee extensors (vastus lateralis (VL), rectus femoris (RF), vastus intermedius (VI), vastus medialis (VM) was measured at mid distance between the lateral condyle of the femur and greater trochanter whereas muscle thickness of plantar flexors (gastrocnemius medialis (GM) and lateralis (GL) and soleus (SOL) was measured at 30% proximal between the lateral malleolus of the fibula and the lateral condyle of the tibia, as previously described [14,15,19], means of an ultrasound apparatus with a 6 cm, 8–10 MHz, linear-array probe (SIEMENS Acuson, P50). Briefly, the transducer was placed parallel to the longitudinal axis of the muscle. The distance between subcutaneous adipose tissue interface and inter-muscular interface was accepted as muscle thickness (Fig 1). The angles between the echo of the deep aponeurosis of the muscle and the interspaces among the fascicles of the muscles was taken as pennation angle. All measurements were analysed in the middle portion of the echographic image.

The cross sectional area (CSA) of the Achilles tendon was captured in the transverse plane at 3 cm proximal to the insertion on the calcaneus; two images were captured with SIMENS software and subsequently analysed with ImageJ software and Photoshop CS5 by the same operator; their mean value was utilized in further analysis.

Achilles tendon length was measured in the longitudinal plane between the calcaneal osteo-tendineous junction and the muscle-tendineous junction of gastrocnemius medialis, by combing multiple images [20,21,22].

The fascicle length across the deep and superficial aponeurosis was manually determined and the relative fascicle length for each muscle was calculated from the ratio of fascicle length and segment length (thigh or shank).

The mean coefficient of variation was 1.7% (range of: 1.65–1.79) for muscle thickness, 2.9% (range of: 2.81–3.00) for pennation angle, 3.5% (range of: 3.43–3.59) for relative fascicle length, 2.3% (range of: 1.94–2.37) for Achilles tendon CSA and 4.0% (range of: 3.89–4.08) for Achilles tendon length (a test-retest on 10 subjects performed by the same investigator).

Finally, the sum of muscle thickness, the mean of pennation angle and relative fascicle length by GL, GM and SOL were used as an indicator of plantar flexor (PF) characteristics, while the sum of muscle thickness, the mean of pennation angle and relative fascicle length by VL, VM, VI and RF were used as an indicator of knee extensor (KE) characteristics.

#### Velocity and kinetic variables analysis

After the muscle-tendon measurements, the athletes (who wore athletic spiked shoes) performed 20 min of warm-up (i.e. running, specific gaits and dynamic stretching) following which they were asked to perform 2 maximal sprints (with a standing start: 3-point start) over a 30 m distance (with 5 min of recovery between trials); a start signal was not provided and the sprinters started when they felt ready. Only the trial with the best acceleration phase (lower sprint time) was analysed.

We asked the subjects to sprint over a longer distance than that we wanted to analyse (30 m instead of 20 m) for two reasons: 1- to avoid the occurrence of a deceleration phase in the last steps of the run and 2- because to fit an exponential function it is necessary to know the amplitude of the signal (e.g. maximal speed). Indeed, the acceleration phase is generally not completed at 20 m but at a distance ≥ 30 m [3,23].

Instantaneous running velocity (*v*) was measured at a sampling rate of 46 Hz with a radar system (Stalker ATS II System) over a distance of 30 m. The radar was placed on a tripod 10 m behind the starting line at a height of 1 m, corresponding approximately to the height of the great trochanter.

The data of running velocity were fitted with a mono-exponential function [24,25] (the velocity onset was ≥ 0.2 m·s^-1^) with a customized Labview (v.10) program:

$${\text{v}(\text{t})=\text{v}}_{\max}\cdot {(1-\text{e}}^{-\text{t}/\tau })$$

where *v* is the modelled running velocity, *v*_*max*_ the maximal velocity reached in the 30 m sprint (i.e. measured by means of the radar gun) and *τ* the time constant of the v vs. t relationship.

The instantaneous forward acceleration (af) and the running distance (d) were calculated from the first derivative and integral respectively:

$${\mathrm{af(t)=ds/dt=(v}}_{\max}{\mathrm{-v}}_{\max}{\mathrm{\cdot (1-e}}^{\;\mathrm{-t/\tau}}\mathrm{))/\tau}$$

$${\mathrm{d(t)=v}}_{\max}{\mathrm{\cdot t-(v}}_{\max}{\mathrm{\cdot (1-e}}^{\mathrm{-t/\tau}}\mathrm{))\cdot \tau}$$

**Fig 2:**
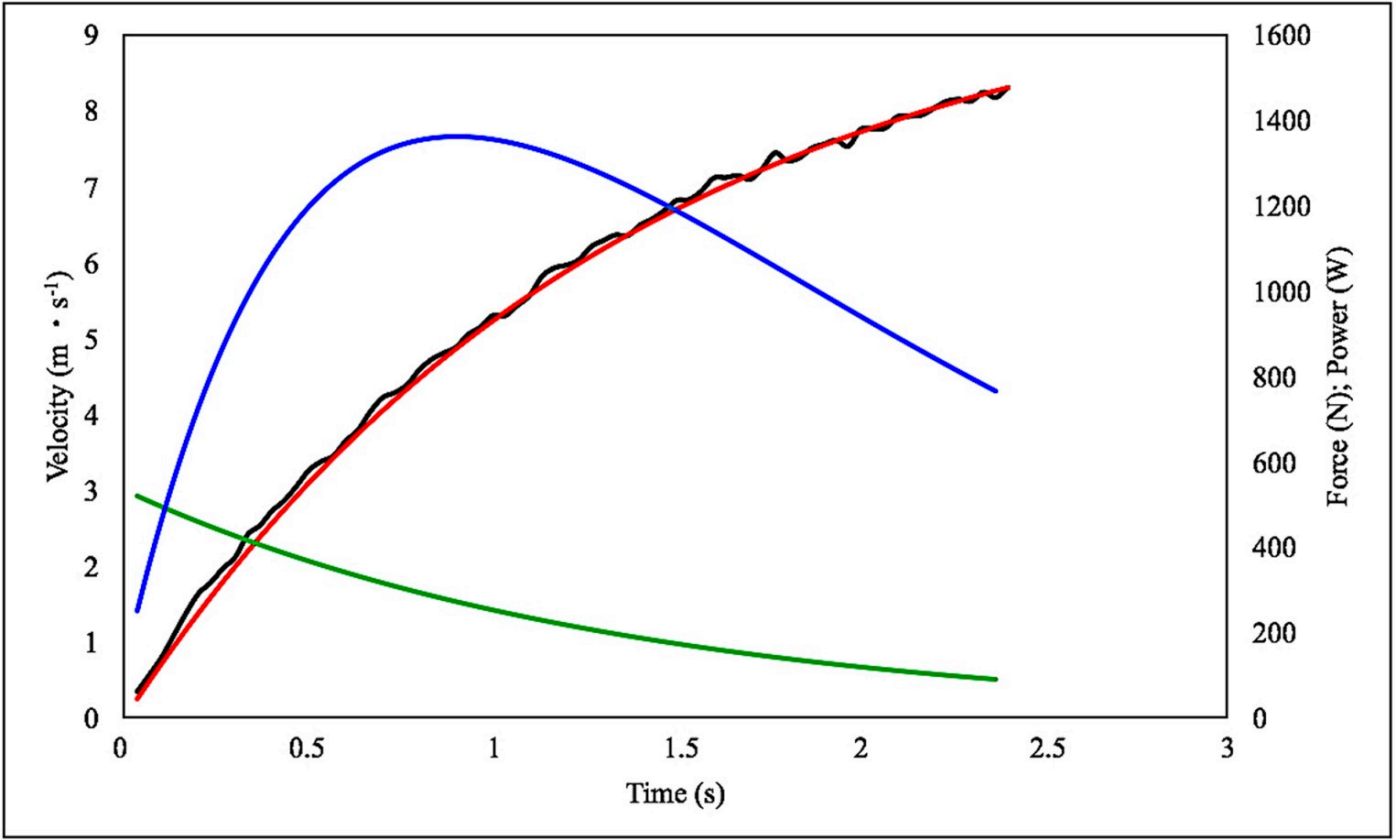
Running velocity, force and power as a function of running time in a typical sprinter. Running velocity (black line); modelled running velocity (red line); force (green line) and power (blue line).

Finally, force (F) and mechanical power (P) were calculated as:

$${F\;=((v}_{\max}{\mathrm{-v}}_{\max}{\mathrm{\cdot (1-e}}^{\mathrm{-t/\tau}}\mathrm{))/\tau )\cdot m}$$

$${\mathrm{P=(((v}}_{\max}-v_{\max}\cdot (1-e^{\mathrm{-t/\tau}}))/\tau )\cdot \mathrm{m)}\cdot {\mathrm{(v}}_{\max}\cdot (1-e^{\mathrm{-t/\tau}}))$$

were *m* is the body mass of subject. The average and peak values of v, F and P were calculated in the acceleration phase only (from 0 to 20 m) (Fig 2). From the velocity, force and power-time curves the mean and peak values for each variable were calculated (v_mean_: mean running velocity; F_mean_: mean force; P_mean_: meanpower; v_peak_: peak running velocity; F_peak_: peak force; P_peak_: peak power). The values of force and power are reported in the paper normalized per kg of body mass.

### Statistical analysis

All variables are expressed as mean ± standard deviations (SD). Relationships between muscle-tendon characteristics and running variables were examined using Pearson correlations. Significant level was set at *P*< 0.05 using SPSS 21.0.

## Results

The mean values and standard deviations of the muscle-tendon parameters are reported in Table 1.

**Table 1:** Architectural characteristics of the analysed muscles. Data are means ± SD. For plantar flexor (PF) and knee extensor (KE) muscles, thickness is the sum of all muscles, while pennation angle and absolute/relative fascicle length are the average of all muscles.

	**Thickness**	**Pennation angle**	**Absolute fascicle length**	**Relative fascicle length**
	**(cm)**	**(°)**	**(cm)**	
**GL**	1.81±0.11	12.0±1.1	7.47±1.09	0.20±0.03
**GM**	2.34±0.19	20.8±1.7	5.87±0.71	0.13±0.03
**SOL**	1.8±0.05	20.1±1.40	5.70±0.77	0.13±0.03
**VL**	2.8±0.13	17.4±2.0	8.19±1.26	0.23±0.03
**VM**	3.51±0.16	11.5±1.5	9.41±1.32	0.40±0.06
**VI**	1.9±0.14	9.0±0.6	8.89±1.22	0.29±0.05
**RF**	2.80±0.17	11.2±1.3	9.14±1.19	0.33±0.03
**PF**	5.99±0.33	17.6±1.3	6.35±0.87	0.15±0.05
**KE**	11.1±0.53	12.8±1.2	8.90±1.26	0.31±0.04
	**CSA (mm**^**2**^**)**	**Length** **(cm)**		
**AT**	72.4±8.7	20.3±4.1		

GL: gastrocnemius lateralis; GM: gastrocnemius medialis; SOL: soleus; VL: vastus lateralis; VM vastus medialis; VI vastus intermedius; RF: rectus femoris; PF: plantar flexors; KE: knee extensors; AT: Achilles tendon.

Muscle thickness of plantar flexors ranged from 1.64 to 2.66 cm, GM showing the largest muscle thickness (range: 2.01–2.66 cm). Pennation angle and relative fascicle length ranged from 10.5 to 23.1° and from 0.08 to 0.26 respectively, GM and GL showing the greater pennation angle and fascicle length (range: 18.4–23.1° and 0.16–0.24, respectively). Knee extensor muscles had a range of 1.77–3.78 cm for muscle thickness and 8.1–22° for pennation angle. VM and VL showed the greater thickness and pennation angle (range: 3.28–3.78 cm and 14.6–22.0°, respectively); the range of relative fascicle length in KE was 0.17–0.50; VM was the longest muscle. Finally, the CSA and length of Achilles tendon ranged from 61.7 and 88.9 mm^2^ and from 13.5 to 26.3 cm, respectively.

Running velocity reached at the end of 20 m (8.88±0.68 m^.^s^-1^) was smaller than at the end of 30 m (9.78±0.71 m^.^s^-1^); thus, in the first 20 m of the sprint running velocity did not reach a steady-state (in all subjects) (v_mean_: 6.31±0.59 m·s^-1^). The values of force were highest at the start of the sprint (F_peak_: 10.02±1.43 N·kg^-1^) and P reached its maximum value 0.97±0.1 s after the start (P_peak_: 26.64±5.99 W·kg^-1^). The average values of force and power, over the entire 20 m distance, were 3.89±0.98 N·kg^-1^ (F_mean_) and 18.23±5.03 W·kg^-1^ (P_mean_). Significant relationships were observed between 100 m PB and v_peak_ (*R* = 0.50; P = 0.045), F_peak_ (*R* = 0.54; P = 0.042), P_mean_ (*R* = 0.65; P = 0.0087), P_peak_ (*R* = 0.65; P = 0.0087) and τ (*R* = 0.60; P = 0.0091) but not with v_mean_ and F_mean_.

The CV% values calculated based on data reported in Table 1 (CV% = SD/mean^.^100) indicate that the variability in the values of velocity (mean and peak over the 20 m distance) is lower (8–9%) than the variability in the mean and peak values of force (N·kg^-1^; 14–25%) and power (W·kg^-1^, 22–28%).

### Relationships between variables

The correlation coefficients (*R*) of the relationships between muscle-tendon characteristics and running variables are reported in Table 2; statistical significance (*P* values) is indicated as well. The mean and peak values of v, F and P increase with muscle thickness and relative fascicle length (*R* = positive) and decrease with pennation angle (*R* = negative) for all muscles and AT. The opposite is true for the time constant (τ) *R* is negative for muscle thickness and relative fascicle length and positive for pennation angle.

**Table 2:** Correlation coefficients (R) and P values (in brackets) between muscle-tendon parameters and running variables for the investigated muscles and for the Achilles tendon.

**Thickness**								
	**PB**	**v**_**mean**_	**F**_**mean**_	**P**_**mean**_	**v**_**peak**_	**F**_**peak**_	**P**_**peak**_	**τ**
**GL**	-0.50^(0.045)^	0.54^(0.042)^	0.38^(NS)^	0.80^(0.000)^	0.85^(0.000)^	0.78^(0.000)^	0.92^(0.000)^	-0.85^(0.000)^
**GM**	-0.55^(0.039)^	0.46^(NS)^	0.28^(NS)^	0.67^(0.005)^	0.69^(0.002)^	0.79^(0.000)^	0.82^(0.000)^	-0.83^(0.000)^
**SOL**	-0.53^(0.043)^	0.46^(NS)^	0.52^(0.044)^	0.78^(0.000)^	0.64^(0.009)^	0.70^(0.002)^	0.82^(0.000)^	-0.82^(0.000)^
**VL**	-0.54^(0.042)^	0.52^(0.044)^	0.29^(NS)^	0.68^(0.003)^	0.71^(0.000)^	0.67^(0.005)^	0.78^(0.000)^	-0.83^(0.000)^
**VM**	-0.68^(0.003)^	0.47^(NS)^	0.25^(NS)^	0.62^(0.009)^	0.62^(0.009)^	0.73^(0.000)^	0.77^(0.000)^	-0.81^(0.000)^
**VI**	-0.56^(0.035)^	0.41^(NS)^	0.20^(NS)^	0.52^(0.044)^	0.53^(0.043)^	0.56^(0.035)^	0.64^(0.009)^	-0.72^(0.000)^
**RF**	-0.61^(0.009)^	0.47^(NS)^	0.27^(NS)^	0.63^(0.009)^	0.63^(0.009)^	0.67^(0.005)^	0.74^(0.000)^	-0.88^(0.000)^
**PF**	-0.52^(0.044)^	0.44 ^(NS)^	0.36^(NS)^	0.76^(0.000)^	0.76^(0.000)^	0.81^(0.000)^	0.89^(0.000)^	-0.92^(0.000)^
**KE**	-0.57^(0.032)^	0.41 ^(NS)^	0.28^(NS)^	0.68^(0.004)^	0.69^(0.002)^	0.73^(0.000)^	0.81^(0.000)^	-0.91^(0.000)^
**AT**	-0.59^(0.027)^	0.51^(0.044)^	0.41^(NS)^	0.78^(0.000)^	0.65^(0.009)^	0.78^(0.000)^	0.88^(0.000)^	-0.90^(0.000)^
**Pennation Angle**								
	**PB**	**v**_**mean**_	**F**_**mean**_	**P**_**mean**_	**v**_**peak**_	**F**_**peak**_	**P**_**peak**_	**τ**
**GL**	-0.64^(0.009)^	-0.57^(0.032)^	-0.35^(NS)^	-0.76^(0.000)^	-0.74^(0.000)^	-0.76^(0.000)^	-0.88^(0.000)^	0.88^(0.000)^
**GM**	-0.70^(0.002)^	-0.47^(NS)^	-0.30^(NS)^	-0.69^(0.002)^	-0.62^(0.009)^	-0.75^(0.000)^	-0.83^(0.000)^	0.91^(0.000)^
**SOL**	-0.61^(0.009)^	-0.52^(0.044)^	-0.26^(NS)^	-0.68^(0.003)^	-0.65^(0.009)^	-0.71^(0.000)^	-0.82^(0.000)^	0.83^(0.000)^
**VL**	-0.33^(NS)^	-0.52^(0.044)^	-0.33^(NS)^	-0.67^(0.005)^	-0.64^(0.009)^	-0.72^(0.000)^	-0.80^(0.000)^	0.66^(0.005)^
**VM**	-0.50^(0.045)^	-0.59^(0.027)^	-0.33^(NS)^	-0.74^(0.000)^	-0.73^(0.000)^	-0.75^(0.000)^	-0.88^(0.000)^	0.87^(0.000)^
**VI**	-0.33^(NS)^	-0.41^(NS)^	-0.19^(NS)^	-0.62^(0.009)^	-0.69^(0.002)^	-0.77^(0.000)^	-0.80^(0.000)^	0.89^(0.000)^
**RF**	-0.47^(NS)^	-0.57^(0.032)^	-0.28^(NS)^	-0.68^(0.003)^	-0.60^(0.009)^	-0.75^(0.000)^	-0.82^(0.000)^	0.85^(0.000)^
**PF**	-0.69^(0.002)^	0.52^(0.044)^	-0.31^(NS)^	-0.74^(0.000)^	-0.80^(0.000)^	-0.77^(0.000)^	-0.88^(0.000)^	0.86^(0.000)^
**KE**	-0.60^(0.009)^	0.51^(0.044)^	-0.33^(NS)^	-0.74^(0.000)^	-0.82^(0.000)^	-0.84^(0.000)^	-0.90^(0.000)^	0.73^(0.000)^
**AT**	/	/	/	/	/	/	/	/
**Relative fascicle length and AT length**								
	**PB**	**v**_**mean**_	**F**_**mean**_	**P**_**mean**_	**v**_**peak**_	**F**_**peak**_	**P**_**peak**_	**τ**
**GL**	-0.63^(0.009)^	0.66^(0.006)^	0.37^(NS)^	0.80^(0.000)^	0.75^(0.000)^	0.80^(0.000)^	0.90^(0.000)^	-0.84^(0.000)^
**GM**	-0.75^(0.000)^	0.58^(0.029)^	0.39^(NS)^	0.75^(0.000)^	0.64^(0.009)^	0.75^(0.000)^	0.86^(0.000)^	-0.88^(0.000)^
**SOL**	-0.53^(0.042)^	0.53^(0.042)^	0.19^(NS)^	0.63^(0.009)^	0.65^(0.009)^	0.77^(0.000)^	0.87^(0.000)^	-0.88^(0.000)^
**VL**	-0.66^(0.006)^	0.49^(0.044)^	0.29^(NS)^	0.72^(0.000)^	0.65^(0.009)^	0.81^(0.000)^	0.88^(0.000)^	-0.88^(0.000)^
**VM**	-0.68^(0.003)^	0.58^(0.029)^	0.39^(NS)^	0.79^(0.000)^	0.69^(0.002)^	0.78^(0.000)^	0.88^(0.000)^	-0.85^(0.000)^
**VI**	-0.73^(0.000)^	0.48^(0.049)^	0.35^(NS)^	0.74^(0.000)^	0.62^(0.009)^	0.77^(0.000)^	0.85^(0.000)^	-0.80^(0.000)^
**RF**	-0.68^(0.003)^	0.58^(0.029)^	0.34^(NS)^	0.75^(0.000)^	0.63^(0.009)^	0.78^(0.000)^	0.88^(0.000)^	-0.85^(0.000)^
**PF**	-0.62^(0.009)^	0.57^(0.032)^	0.31^(NS)^	0.79^(0.000)^	0.67^(0.005)^	0.76^(0.000)^	0.88^(0.000)^	-0.84^(0.000)^
**KE**	-0.66^(0.006)^	0.53^(0.042)^	0.35^(NS)^	0.72^(0.000)^	0.64^(0.009)^	0.78^(0.000)^	0.87^(0.000)^	-0.80^(0.000)^
**AT**	-0.70^(0.002)^	0.48^(0.049)^	0.32^(NS)^	0.71^(0.000)^	0.62^(0.009)^	0.75^(0.000)^	0.83^(0.000)^	-0.88^(0.000)^

GL: gastrocnemius lateralis; GM: gastrocnemius medialis; SOL: soleus; VL: vastus lateralis; VM vastus medialis; VI vastus intermedius; RF: rectus femoris; PF: plantar flexors; KE: knee extensors; AT: Achilles tendon; PB: 100 m personal best time; v: running velocity; F: force; P: power; τ: time constant of the v vs. t relationship; NS: no significant relationship; 0.000 = P < 0.001.

**Fig 3:**
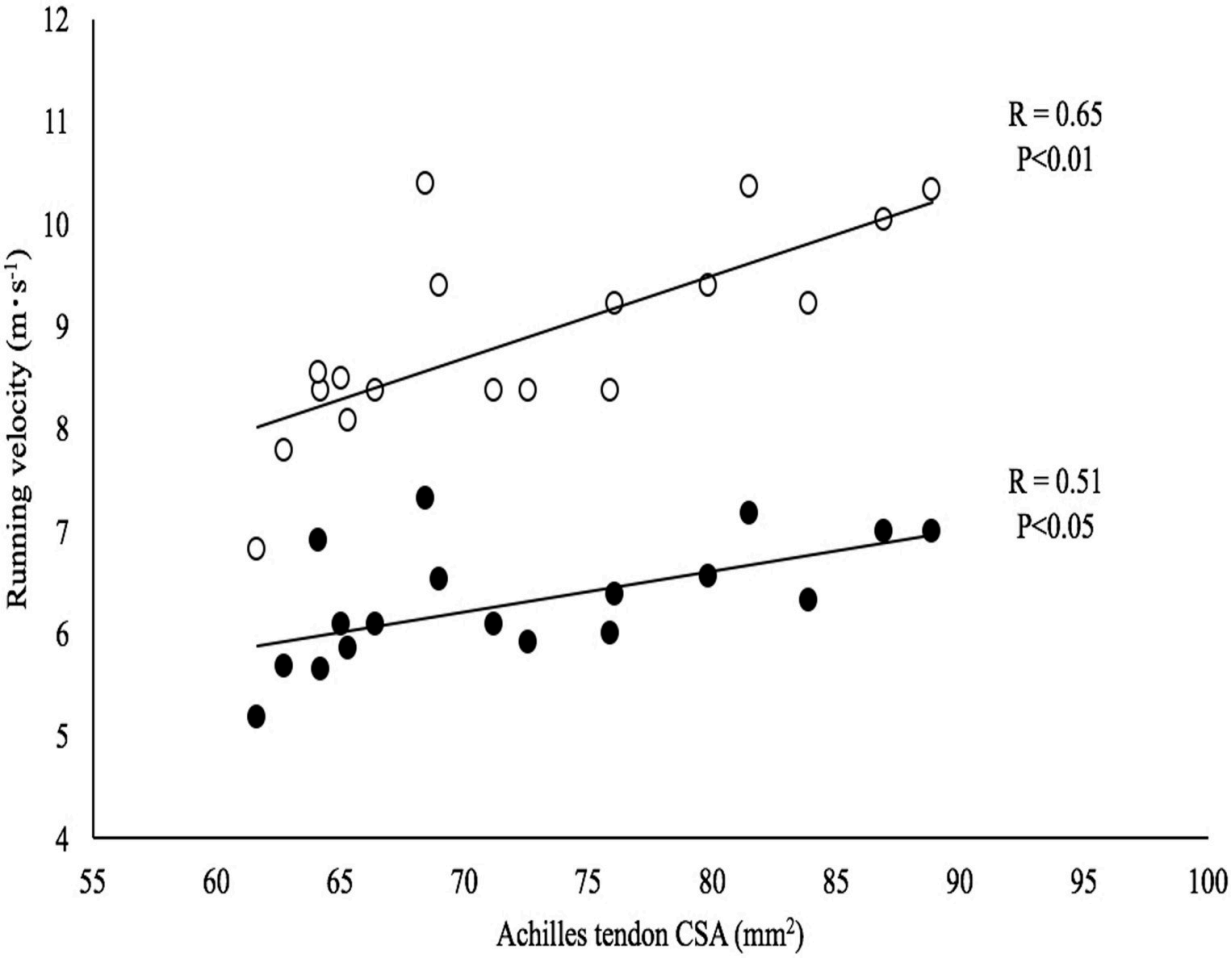
Running velocity as a function of the Achilles tendon cross sectional area (CSA). Mean (full circles) and peak (open circles) running velocity.

The effects of inter-subject differences in Achilles tendon CSA on v_mean_, v_peak_ are reported in Fig 3 as an example of these relationships. This figure shows that a larger CSA of the Achilles tendon is associated with greater values of v_mean_ and v_peak_ and that the correlation coefficient (and the level of significance) of these relationships is lower for v_mean_ than for v_peak._

Indeed, as can be seen by inspection of Table 2, correlations with v_mean_ are relatively weak compared to those with v_peak_ and τ; better correlations (larger *R* and lower *P*) are observed between muscle-tendon characteristics and peak values of force and power than with the corresponding mean values. The strongest correlation between muscle-tendon proprieties and running variables is observed when considering peak power output (P_peak_) or the time constant (τ).

## Discussion

In this study the relationship between muscle-tendon parameters and force/power production during the sprint running acceleration phase (in the first 20 m) was investigated; we observed that muscle thickness and relative fascicle length, as well as Achilles tendon CSA and length, are positively correlated with sprint running performance (with values of v, F and P) while pennation angle is negatively correlated with it.

Force and power were not directly measured but calculated based on values of horizontal speed. This is a limitation of this study (see critique of methods); however, this analysis allowed us to get a better insight on the determinants of sprint performance in comparison with previous studies where only the average (horizontal) speed over the 100 m distance was taken into consideration. Indeed, as indicated in the Introduction, relatively weak relationships (or no relationship) between muscle-tendon characteristics and best times over the 100 m distance were reported in previous studies [12,14,15,18]. Our findings indicate that the correlation coefficients of the relationships between muscle-tendon parameters and sprint performance largely improve when peak values of v, F and P in the acceleration phase are considered (R range: 0.62–0.92), peak power and τ being the parameters best correlated with muscle-tendon proprieties (R range: 0.73–0.96). In this study we did not ask our sprinters to run the entire 100 m distance; their current 100 m PB times were utilized instead. This is a limitation of this study; however, these data confirm that the correlation coefficients are “weaker” in this case, and similar to those referring to v_mean_ in the acceleration phase: R range: 0.41–0.61).

In the following paragraphs the parameters investigated in this study will be discussed in comparison with the values reported in the literature.

### Sprint variables

Running velocity increases as a function of time, while horizontal force production decreases, as previously reported in the literature [3,19,26]. Our P_mean_ values (1305±420 W; 18.23±5.03 W^.^kg^-1^) are similar to those reported by others during a sprint running acceleration phase (e.g. Slawinski et al. [7]: 15.3±03.3 W^.^kg^-1^; Monte et al. [26]: 17.2±3.63 W^.^kg^-1^). Moreover, our τ values (1.40±0.04 s) are close to those reported by others in subjects with similar characteristics (1.34±0.7 s, e.g. Samozino et al. [19]).

Significant relationships were observed in this study between 100 m PB and v_peak_, F_peak_, P_mean_, P_peak_ and τ but not with v_mean_ and F_mean_. These results are in agreement with data reported by Slawinski et al. [7] who indicate that 100 m personal best times are better related to the average power output (*R* = -0.69) in the first 20 m of a 100 m race than to the average running velocity over this distance (*R* = -0.29). The correlation coefficient reported in this study for P_mean_ (*R* = -0.65) is similar to the one reported by these authors.

Our results show that the variability (CV%) in the values of mean and peak velocity over the 20 m distance (8–9%) is lower than the variability in the mean and peak values of force and power (15–30%). This indicates that F and P are better indicators (than v) of the inter-subject variability in the acceleration phase of a sprint.

### Muscle-tendon variables

Our data of muscle thickness, pennation angle, fascicle length and Achilles tendon length and CSA are comparable to data reported in literature in subjects with similar characteristics [14,16,20,22].

Our results suggest that muscle thickness is positively correlated with power production during sprint running in agreement with previous studies that highlighted a relationship between muscle thickness and power output [27] and in agreement with Kubo et al. [16] who reported that muscle thickness of knee extensor and plantar flexors is significantly greater in sprinters than in controls. Miyake et al. [18] reported no relationship between 100 m personal best time and the quadriceps CSA; we suggest that this finding, rather than to a lack of relationship between CSA and sprint abilities, which should be expected on theoretical basis, could be attributed to the fact that “average speed”, instead of power output, was taken into account.

An increase in muscle thickness (e.g. as a result of a strength training protocol) is associated to a greater force production capacity of the muscle [9]. Hence, a muscle with a larger muscle thickness is expected to exert a larger force on the ground; accordingly, the acceleration ability of the athlete is expected to improve, due to the positive relationship between force production and acceleration performance [5,28].

Another important aspect of muscle architecture in relation to sprint running performance regards muscle length. As suggested by Abe et al. [20], a greater fascicle length would confer greater velocity capacity, during identical tendon excursion, in the sprint acceleration phase. This because a fiber containing more sarcomeres in series would contract at a greater velocity than a fiber containing less sarcomeres in series; as a result, power production is expected to be greater in the former case, as well as sprint performance [9]. Our data support this theoretical background, in fact, we observed a strong positive correlation between (relative) fascicle length and mechanical power production (see Table 2), in agreement with other studies [6,9].

Finally, because pennation angle “geometrically” depends on muscle thickness and fibre length, it is also expected to play an important role on sprint running performance. This was previously observed by Kumagai et al. [14] who reported a positive relationship between pennation angle and 100 m PB; accordingly, we observed a negative relationship between pennation angle and sprint velocity. It is possible that a fiber with a greater pennation angle operates closer to its optimum length and, based on the length-tension relationship, is thus able to generate more force [29]. In fact, pennation affects the relative velocity of fibre shortening. To generate a given degree of shortening in the whole muscle, individual muscle fibres in a pennate muscle have to shorten further than those in a fusiform muscle [30]. Consequently, for any given velocity of whole muscle movement, the more pennate the muscle the closer to their maximum velocity the individual fibres are working. However, as suggested by Azizi et al. [30] the fibre rotation that occurs during a contraction contributes to increase the shortening velocity of the whole muscle by allowing the muscle to function at a higher gear ratio. Therefore, this mechanism may potentially increase mechanical power production for a given overall muscle shortening speed in pennate compared to non-pennate muscle.

An important result of our study is the strong positive relationship between mechanical power output during a sprint and Achilles tendon CSA and length. Indeed, tendon morphological characteristics could influence its mechanical proprieties (e.g. its stiffness). As an example, Bohm et al. [31] showed that a higher stiffness and a larger CSA of the Achilles tendon could have a positive effect on running performance, influencing the stretch-shortening cycle and Lai et al. [12] showed, based on experimental data and computational modeling, that tendon elasticity plays an important role in enhancing power output at the start of a maximal sprint. A greater tendon CSA (e.g. as a result of training adaptation) should thus allow the runner to withstand a larger mechanical stress (allowing the sprinter to reach higher running speeds). Indeed, we observed that the faster runners are those with the greater Achilles tendon CSA, as previously reported by others [22].

### Critique of methods

Radar devices estimate the displacement of the body center of mass based on the displacement of the subject’s lower back. During a sprint start, the BCoM position changes in relation to the lower back position; moreover, BCoM rises vertically while the radar or laser tripod height does not change [24]. Therefore, based on these methods it is possible to calculate, using the fundamental laws of dynamics, horizontal force but not its vertical component; moreover, the fluctuations of velocity at each step (both along the vertical and horizontal axes) are not taken into account. Hence, even if the radar technology is considered a valid and reliable method for measuring sprinting speed [32], it is important to remember that force and power values determined with this method are underestimated compared to the real values.

It must be pointed out that, in a sprinting task, the aim is to cover in the shortest possible time a given horizontal distance; therefore, we think that the results of this study are meaningful and of interest even if the values of force were calculated (and not measured directly) in the forward direction only. Further studies should ascertain whether the strong correlations reported in this study between F and P and muscle-tendon characteristics still hold when F and P are directly measured (e.g. by means of force platforms).

## Conclusion

The findings of this study improve our understanding of the interplay between muscle-tendon characteristics and sprint performance: the stronger correlations are observed between muscle-tendon parameters and peak power output and this suggests that considering/measuring this parameter: i) could allow to better understand the relationship between musculoskeletal morphometry and sprinting ability; ii) could allow to better understand the interplay between all these factors in determining sprint performance and iii) could increase the possibility to detect significant training induced changes.

These findings have implications not only for track and field sports but for all team sports involving sprinting that rarely cover more than 20 m in a straight line (e.g. rugby, soccer and football). This study provides information about how the architectural characteristics of knee extensors, plantar flexors and Achilles tendon contribute to power generation during a sprint running acceleration phase. These data show that the relationship between muscle tendon parameters and sprinting performance is particularly evident in the sprint acceleration phase were the largest forces are exerted and peak power is attained.
